# RNA FRABASE 2.0: an advanced web-accessible database with the capacity to search the three-dimensional fragments within RNA structures

**DOI:** 10.1186/1471-2105-11-231

**Published:** 2010-05-06

**Authors:** Mariusz Popenda, Marta Szachniuk, Marek Blazewicz, Szymon Wasik, Edmund K Burke, Jacek Blazewicz, Ryszard W Adamiak

**Affiliations:** 1 Institute of Bioorganic Chemistry, Polish Academy of Sciences, Poznan, Poland; 2 Institute of Computing Science, Poznan University of Technology, Poznan, Poland; 3 Poznan Supercomputing and Networking Centre, Poznan, Poland; 4 Automated Scheduling, Optimisation and Planning Group, School of Computer Science, University of Nottingham, Nottingham, UK

## Abstract

**Background:**

Recent discoveries concerning novel functions of RNA, such as RNA interference, have contributed towards the growing importance of the field. In this respect, a deeper knowledge of complex three-dimensional RNA structures is essential to understand their new biological functions. A number of bioinformatic tools have been proposed to explore two major structural databases (PDB, NDB) in order to analyze various aspects of RNA tertiary structures. One of these tools is RNA FRABASE 1.0, the first web-accessible database with an engine for automatic search of 3D fragments within PDB-derived RNA structures. This search is based upon the user-defined RNA secondary structure pattern. In this paper, we present and discuss RNA FRABASE 2.0. This second version of the system represents a major extension of this tool in terms of providing new data and a wide spectrum of novel functionalities. An intuitionally operated web server platform enables very fast user-tailored search of three-dimensional RNA fragments, their multi-parameter conformational analysis and visualization.

**Description:**

RNA FRABASE 2.0 has stored information on 1565 PDB-deposited RNA structures, including all NMR models. The RNA FRABASE 2.0 search engine algorithms operate on the database of the RNA sequences and the new library of RNA secondary structures, coded in the dot-bracket format extended to hold multi-stranded structures and to cover residues whose coordinates are missing in the PDB files. The library of RNA secondary structures (and their graphics) is made available. A high level of efficiency of the 3D search has been achieved by introducing novel tools to formulate advanced searching patterns and to screen highly populated tertiary structure elements. RNA FRABASE 2.0 also stores data and conformational parameters in order to provide "on the spot" structural filters to explore the three-dimensional RNA structures. An instant visualization of the 3D RNA structures is provided. RNA FRABASE 2.0 is freely available at http://rnafrabase.cs.put.poznan.pl.

**Conclusions:**

RNA FRABASE 2.0 provides a novel database and powerful search engine which is equipped with new data and functionalities that are unavailable elsewhere. Our intention is that this advanced version of the RNA FRABASE will be of interest to all researchers working in the RNA field.

## Background

A deep knowledge of complex three-dimensional folds of RNA structures is essential to understand the range of their biological functions. Both the Protein Data Bank (PDB) [1] and the Nucleic Acids Database (NDB) [2] hold an indispensable collection of RNA structures. This reservoir has dramatically increased in the past few years and it now contains over 1700 tertiary RNA structures, both, in the form of isolated molecules as well as RNA-protein and ligand complexes. Deposited files provide atomic coordinates and a wealth of structural data for further processing and analysis. However, the number of such files is still much smaller than the protein structures available (about sixty thousands).

Several dedicated programs, web-accessible servers and databases have been proposed for processing and analysis of the PDB files (c.f. [3]) to study three-dimensional RNA structure. Among them, two particular programs are of general use and of specific importance to our work: 3DNA [4] and RNAView [5]. The first program calculates a complete set of rigid-body parameters that describe the detailed geometry of double helices extracted from the tertiary RNA structures. The second one finds all base pairs and multiplets in the RNA structure and offers the classification of canonical and non-canonical base-pairs. Both programs have been implemented in web-servers [2,6].

One of the most difficult issues in mining available structural data is the search of RNA structural motifs and fragments in a conformational space. Until now, only a small number of methods have been developed to cope with this kind of data exploration using various searching patterns. Initial approaches have been based on a representation of the RNA conformational space which reduces to pseudotorsional angles η and θ (PRIMOS [7]) or dihedral angles (DIAL [8]). Recently, a more general approach has focused on systematic description and analysis of dihedral angles and has been exploited by the RNA Ontology Consortium [9]. Another idea, which was used in the FR3D program [10], is based upon searching for RNA motifs on the basis of a complex pattern containing annotations of base-base and base-phosphate interactions. Finally, a program called ARTS [11] has used a 3D alignment approach based on templates covering the RNA backbone atom coordinates.

Recently, we have published RNA FRABASE 1.0 [12], a web-accessible database with an engine for automatic search of the 3D fragments within PDB-derived RNA structures. The search pattern served by this tool is composed of RNA sequence(s) and/or secondary structure(s) given in the dot-bracket format. It has used an algorithm based on regular expressions and a pattern-matching method. This has enabled very fast search. The output list of three-dimensional RNA fragments, having the defined query-matching canonical secondary structure, provides access to their coordinates and can be directly downloaded and visualized at the client site. It should be emphasised that the basic searching principles of RNA FRABASE 1.0 have been recently adopted in the FASTR3D web server [13]. However, in several aspects, this server has distinct limitations when compared with our system.

Our approach has the advantage of interconnecting the search engine with the dedicated database. Although, PDB and NDB hold a complete collection of RNA structures and provide a wealth of structural data, several dedicated RNA databases have been proposed to facilitate the RNA tertiary structure analysis on different levels and from different points of view. The SCOR database [14], now discontinued, has surveyed three-dimensional RNA motifs, offered access to over eight thousand RNA structural elements, mostly internal and hairpins loops, and focused on tertiary interactions. Other databases have been focused on various RNA motifs, among them: RNA junction loops (RNAJunction [15]), base pairs and multiplets (BPS [16]), fragments (RNA FRABASE [12], FASTR3D [13]) and residues (MODOMICS [17]).

In this paper, we describe RNA FRABASE 2.0 which has been equipped with powerful new search algorithms and data mining functionalities. It provides an intuitionally operated web server platform for proficient, very fast exploration of three-dimensional RNA structures and their multi-parameter conformational analysis. Due to frequent RNA FRABASE 1.0 user requests, the new library of RNA secondary structures (with graphics) has been made available. An instant visualization of the tertiary RNA structure is provided.

## Construction and content

**Figure 1:**
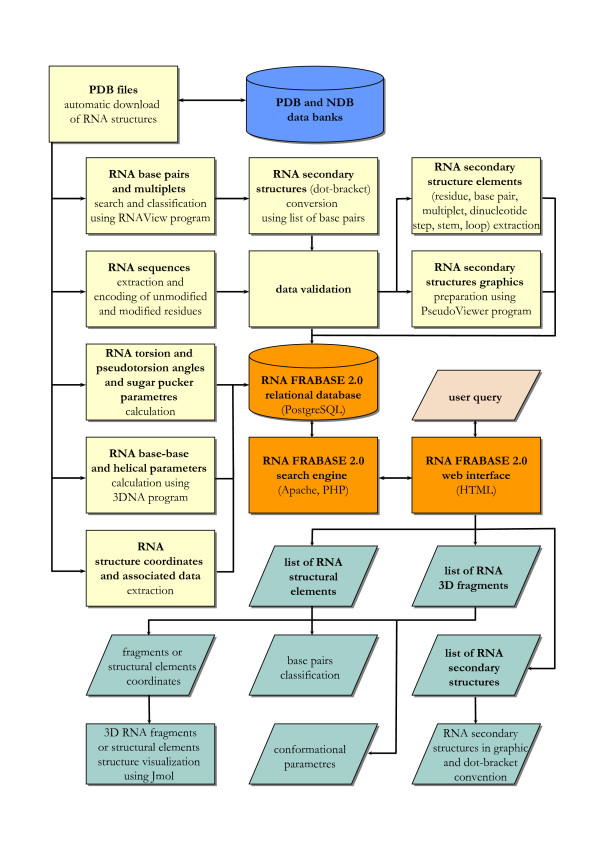
**Data flow in RNA FRABASE 2.0**. The overall data encoding idea that underpins RNA FRABASE 2.0 (illustrated by the sections of the diagram in orange) is based on the information deposited in PDB and NDB (shown by the cylinder in blue), our own scripts and external applications (denoted by the rectangles in yellow). The search algorithms output a broad range of structural information (represented by the parallelograms in turquioise).

The core of RNA FRABASE consists of the database, the search engine and the web interface. All the actions undertaken by the user involve these three key components of the system. In addition, a number of satellite programs and routines are responsible for computation and the processing of the structural information which is supplied to the database (Figure 1). This new version of RNA FRABASE represents a series of major improvements and innovations which are outlined in the following paragraphs.

### Database

RNA FRABASE 2.0 stores a large collection of structural data about RNA molecules, in both their isolated and complexed form, all of which is derived from their PDB-deposited atom coordinates. It is automatically updated once a month. A complete set of models (not just the first one), collected in PDB for Nuclear Magnetic Resonance (NMR) and X-ray structures is processed and stored in the database. In total, as of December 2009, the database contains 1565 RNA structures. This represents about 88% of the 1770 deposited in the Protein Data Bank, including all the most important ones. Small structures (without at least one canonical base pair) or those built from non-natural nucleotide residues are not collected. Key statistical information about the contents of the database with respect to the experimental method used are presented in Table 1.

**Table 1:** RNA FRABASE 2.0 database statistical information by experimental method.

Experimental method	structures	models	strands	residues	residues without coordinates (%)
X-ray	1099	1110	2571	680562	20620 (3.03%)

NMR	424	5013	6259	135001	417 (0.31%)

Electron microscopy	41	41	138	51108	763 (1.49%)

Other	1	1	1	49	0

All methods	1565	6165	8969	866720	21800 (2.52%)

RNA sequences, secondary structures, the PDB header data (identification codes, experimental method used, resolution, PDB deposition date, number of models) and the NDB identification codes represent the core of RNA FRABASE 2.0. The first two of these are necessary to perform automatic pattern-based search of the 3D RNA fragments. A pattern within the database is composed of the sequence(s) and/or secondary structure(s) coded in dot-bracket format extended to hold multi-stranded structures. Since, the quality of the PDB-derived secondary structures affects the search results, it is important to hold the negative errors that occur in the structure description. Therefore, the dot-bracket convention used in RNA FRABASE 2.0 has been extended to cover RNA residues which are missing from the PDB coordinate section. Now, dot-bracket encoded RNA secondary structures contain a sign "-" to highlight these residues. This change has eliminated the search errors that were often induced by the omission of certain coordinates, mainly in X-ray structures.

**Figure 2:**
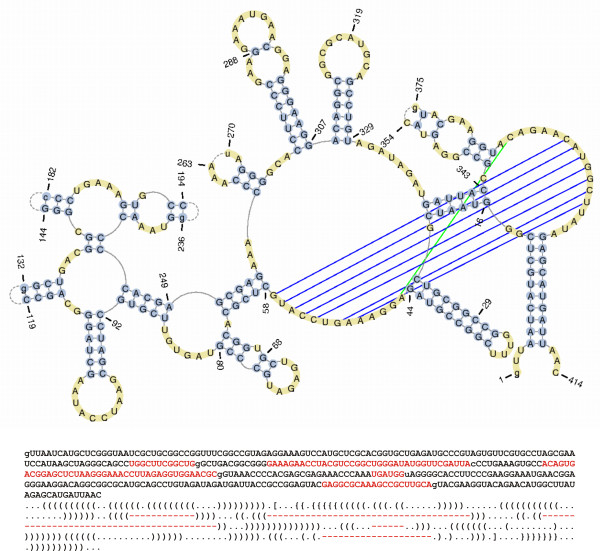
**An example of the RNA secondary structure which includes residues with missing coordinates (2A64)**. The figure illustrates a graphic image of the secondary structure (top), the sequence (middle) and dot-bracket representation of the secondary structure (bottom) for he 2A64 RNA molecule. The residues missing from the PDB file coordinate section are marked in red.

This modification has prompted us to prepare a new library of RNA secondary structures which is now available to the user under the "Secondary structures" menu. Although similar libraries are provided by some other databases, e.g. RNA STRAND [18], only RNA FRABASE 2.0 presents, at the secondary structure level, information about residues whose coordinates are missing from PDB files (see Figure 2 for example) and which are connected to the tertiary structure parameters.

**Figure 3:**
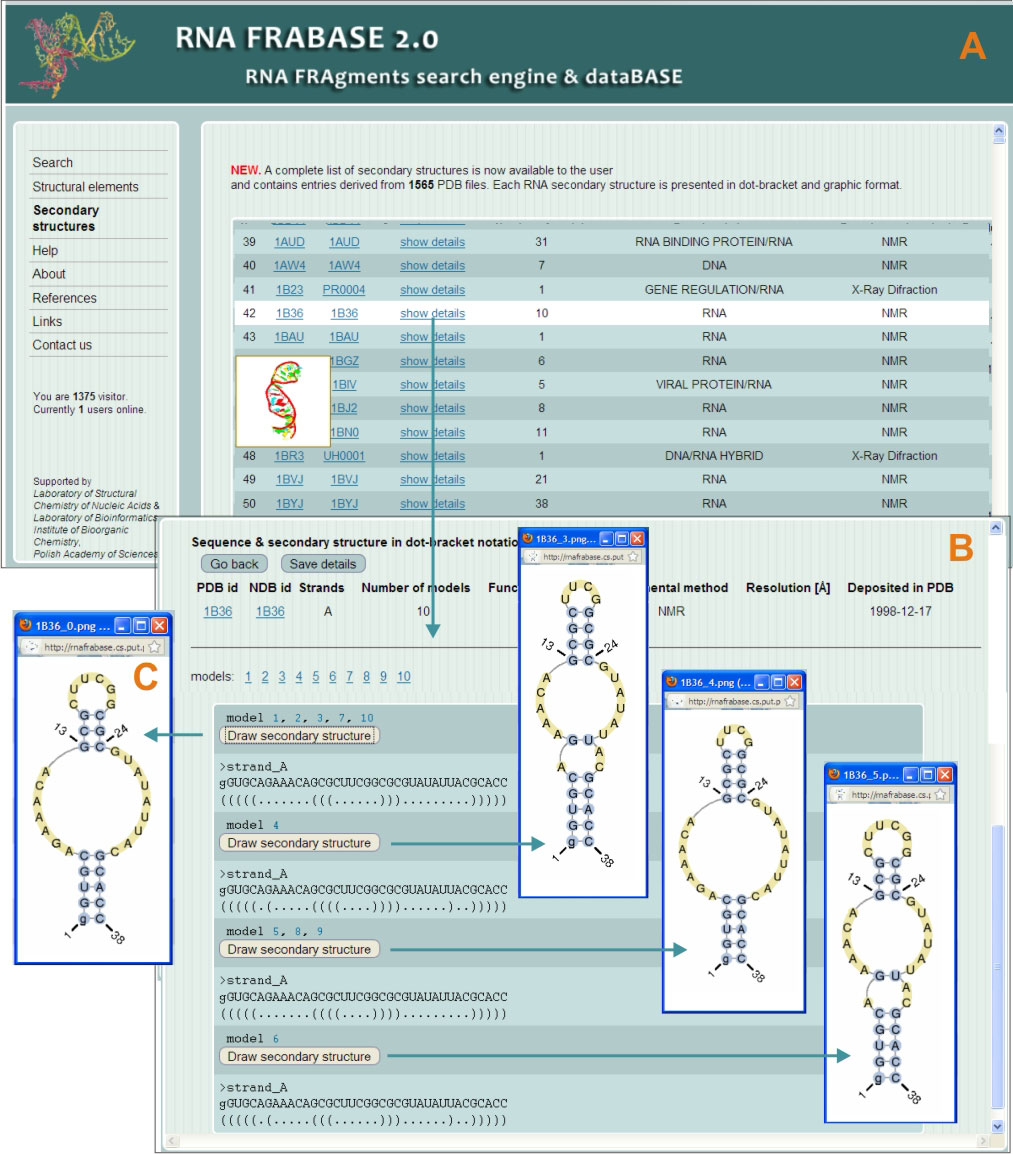
**Accession to the database of RNA secondary structures given in dot-bracket and graphic format**. Here we see a range of RNA FRABASE 2.0 interface snapshots concerning "Secondary structures" menu. Panel A shows the front page window, Panel B illustrates the 'Details' window for the selected structure and Panel C represents a visualization of the selected secondary structure model.

For the convenience of the user, each RNA secondary structure can be visualized on the spot (see Figure 3). We have applied a new routine which enables us to draw even very large and complex RNA secondary structures immediately. It first processes structural data (sequence and secondary structure), eliminating the information about the pseudoknots, missing residues, modified residues, etc. For such processed data, the PseudoViewer software [19] is launched to generate the view of a structure as a postscript file. Finally, this file is processed again to restore the information removed in the first step. The above procedure is performed once for each structure, when it is supplied to the database. Graphic representations are stored in the database and can be immediately viewed upon the request of the user. Figure 2 presents an example of a graphic representation of the RNA secondary structure generated by the above mentioned routine.

Apart from the core data, RNA FRABASE 2.0 contains:

(i) a collection of atom coordinates of unmodified and modified nucleotide residues occurring in RNA structures;

(ii) a description of torsion angles and sugar pucker parameters, including values for the pseudotorsion angles, η (defined by atoms C4'_(n-1)_-P_(n)_-C4'_(n)_-P_(n+1)_) and θ (defined by atoms P_(n)_-C4'_(n)_-P_(n+1)_-C4'_(n+1)_) calculated by our own scripts;

(iii) base-base and inter base pair parameters;

(iv) base pair location, i.e. information specifying whether one or both bases of the pair belong to the resulting fragment;

(v) a complete classification of canonical and non-canonical base pair types, in both Westhof's and Saenger's notation and information about multiplets;

(vi) information about original residue and chain numbering.

Although some of this information might be obtained from the other sources, we have collected it together to provide the user with the widest possible set of structural features of the 3D RNA fragments which are of interest and to use them as structural filters in the search.

Moreover, in comparison to version 1.0, the RNA FRABASE 2.0 database has been extended to increase the effectiveness of searching for small 3D fragments. Now, the database stores additional information about basic structural elements, such as residues, base pairs, multiplets, dinucleotide steps, stems and loops (apical, bulge, internal and n-way junctions). They are extracted from the sequence and secondary structure which is encoded in dot-bracket notation by our own scripts and loaded to the database records (Table 2).

**Table 2:** Statistical information about the structural elements stored in RNA FRABASE 2.0.

Type of structural element	Number of structural elements in all models of the deposited structures	Number of structural elements in the 1^st ^model of the deposited structures
Residue	866720	742632

Base pair	505900	443962

Multiplet:		
-triplet	25669	23645
-quadruplet	3806	3523
-quintuplet	97	97

Dinucleotide step	332564	270986

Stem	50873	42297

Loop:		
-hairpin	21115	16650
-internal/bulge	34204	29193
-3-way junction	4117	4077
-4-way junction	1975	1944
-5-way junction	1196	1196
-6-way junction	175	175
-7-way junction	214	214
-8-way junction	23	23
-9-way junction	3	3
-10-way junction	9	9
-11-way junction	23	23
-12-way junction	5	5
-14-way junction	1	1

Individual residues, which can be queried in RNA FRABASE 2.0, are coded in one-letter format. Unmodified RNA monomer residues are encoded in capital letters, i.e. A, C, G, U. All modified RNA and non-RNA units, like DNA and 5'-dephosphorylated residues are represented by small letters {a, c, g, u} giving the closest analogy to parent nucleoside. For each residue, conformational parameters are available. More detailed information about modified residues can be found in the MODOMICS database [17].

Base pairs are another structural elements which can be searched in RNA FRABASE 2.0. The canonical and non canonical base pairs are classified using the RNAView program [5] and their base-base and inter base pair parameters are calculated using the 3DNA program [4]. The higher-order base interactions are identified by applying RNAView and placed in the multiplet records. Their number is similar to that recently reported in the BPS database [16]. Dinucleotide steps involve two canonical base pairs and they are characterized by the base-base and inter base pair parameters, which have been calculated using the 3DNA program [4].

**Figure 4:**
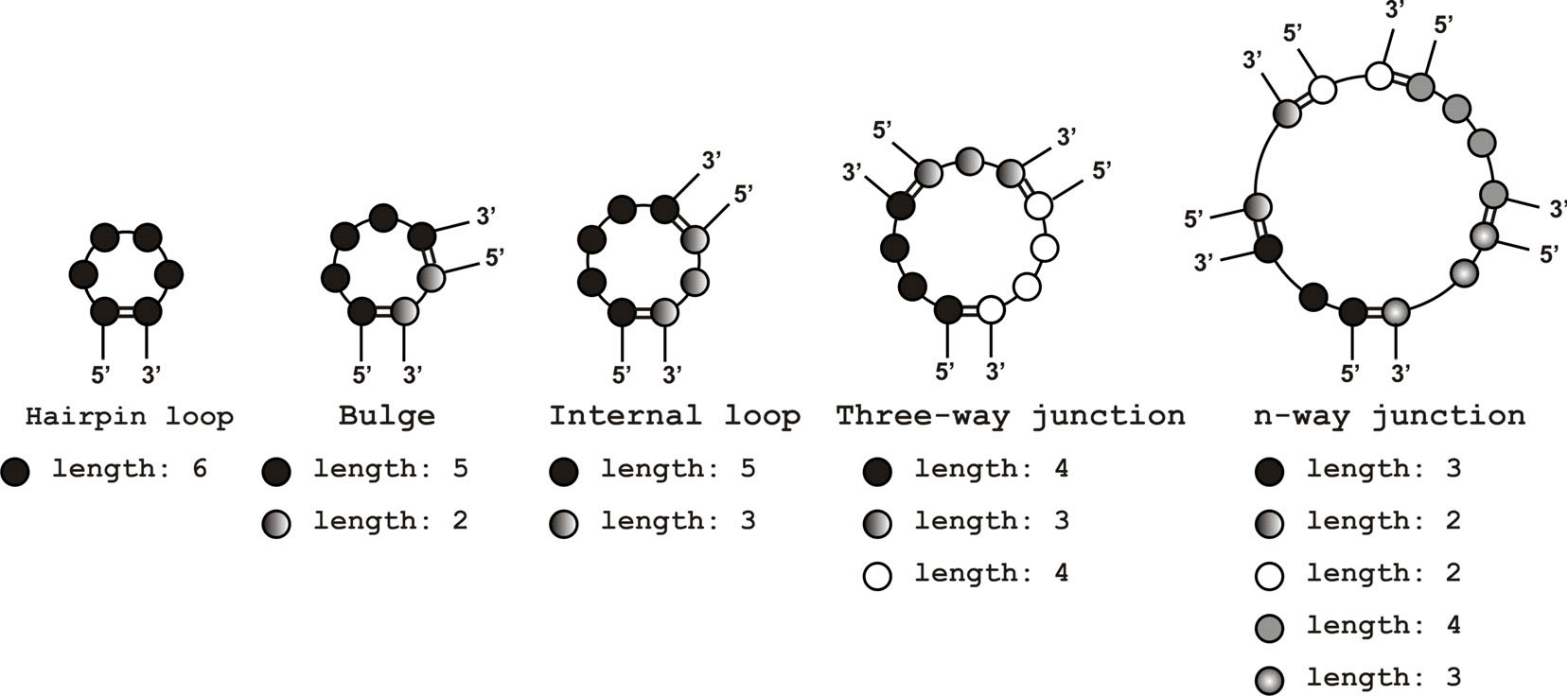
**Example loop structures stored in RNA FRABASE 2.0 database**. Each residue within the RNA loop is represented by a circle. Different shades of gray enable us to distinguish between single strands within the same loop. Two circles connected by a double line represent a closing base pair.

Stems and loops (apical, bulge, internal and n-way junctions) are identified and extracted by our own scripts. The stem segments contain only canonical base pairs. The loop structure is composed of one- (hairpin apical loops), two- (internal loops and bulges) or n-strands (n-way junctions) closed, respectively, by one, two or n canonical base pairs (see Figure 4 for an example). This is an alternative approach to that offered in the RNAJunction database [15] which considers both canonical and non-canonical base pairs closing the loop.

All the structural elements described above are served by a separate search algorithm. Our extension of the database and the design of the second algorithm enabled us to remove the search threshold set in RNA FRABASE 1.0 to block the listing of highly populated fragments (i.e. those smaller than four residues).

### Search engine

The RNA FRABASE 2.0 engine has been designed to search for user-specified single- or multi-stranded RNA fragments. The number of strands composing a fragment is unlimited. The user provides an input pattern describing those strands in the input box of the "Search" page or selects an appropriate tab sheet in the "Structural elements" page. To ensure effective performance, one should follow the RNA FRABASE format, which is described in detail within our "Help" pages. Input data validation procedures have been also implemented for user convenience.

The search engine in version 2.0 is based upon two efficient algorithms. The first algorithm processes the queries inserted through the "Search" page. The basic underpinning idea is the same as for the algorithm implemented in RNA FRABASE 1.0, but it has been updated to hold the extended searching pattern and new filtering options. The second algorithm has been introduced to search for basic structural elements within the "Structural elements" page. Its application has allowed us to disable the threshold, set in RNA FRABASE 1.0 to block the search for small, highly populated, structural fragments. Consequently, the searching potential of RNA FRABASE has been significantly expanded.

**Figure 5:**
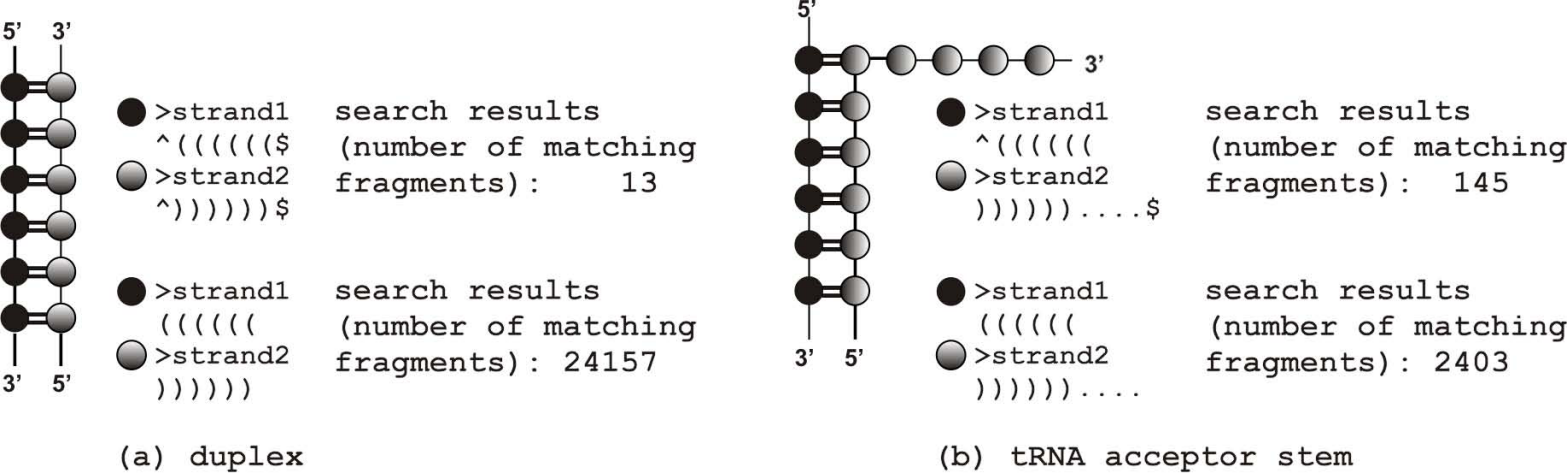
**Example of the use of the wildcard characters "^" and "$" in the query**. The figure shows two example structures: (a) RNA duplex and (b) tRNA acceptor stem. The input pattern used to run search algorithm and the number of matching fragments are provided for each example. Residues, base pairs and strands are marked by employing the same convention as that used in Figure 4.

Wildcard characters are now allowed in the definition of the query pattern. They include: "^" (up arrow), which determines search for the 5'-end fragment only, "$" (dollar symbol) determining search for the 3'-end fragment only and "?" (question mark) which stands for any residue which might be paired, unpaired or missing. The "^" and "$" wildcards can be placed only at the beginning or at the end of the strand both within a single or multi-strand fragments of user interest (see Figure 5 for examples). The "?" wildcard can be allocated to any place but only for single strand fragments. In the case of multi-strand fragments, this wildcard operates only on the 5'- and 3'-ends of the respective strand.

New advanced options can be outlined as follows:

(i) include all models of the structure;

(ii) add through-space interactions;

(iii) enable strand shift operation.

**Figure 6:**
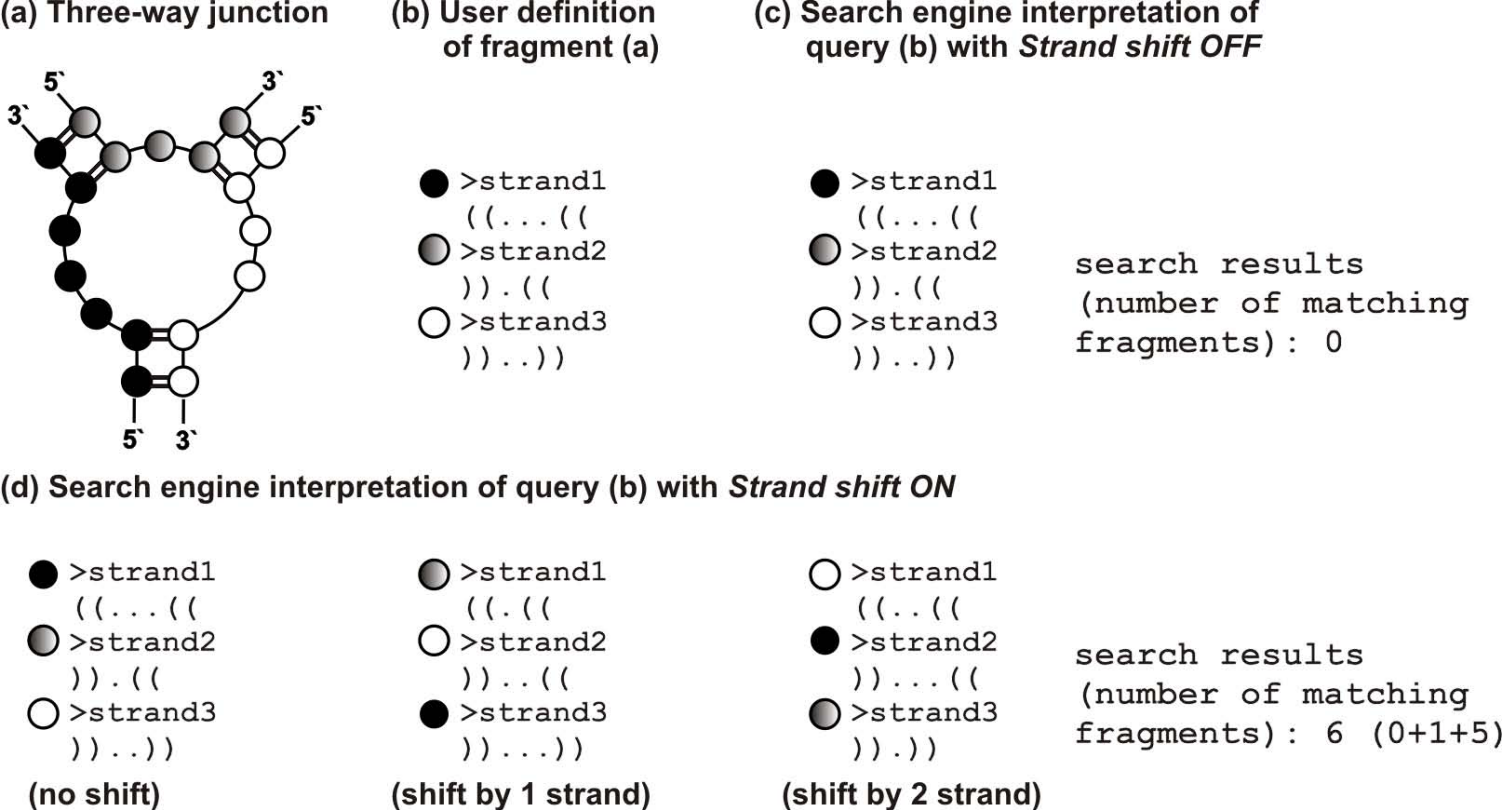
**The influence of the strand shift operation on 3D fragment search results**. The figure illustrates the search for the RNA three-way junction (a). It is defined by the input pattern shown in section (b) which differs when the strand shift operation is disabled (c) or enabled (d). If the strand shift is enabled, then the RNA FRABASE engine searches for fragments which match with one of the three patterns shown in (d).

These are available from both, the "Search" and "Structural elements" menus and improve the requested 3D RNA fragment specification. The first option, when checked, allows the search of the 3D fragment within all the models available basically for the NMR structures. When it is not checked, only the first model of each deposited structure is considered by the RNA FRABASE engine during the search process. The second option has been introduced to support the selection of fragments, such as bulges or loops, which interact through space with other RNA strands or fragments. The third option has been introduced to search for di- and multi-stranded fragments (c.f. [20]) and is based on a concept which is analogous to a bitwise shift operator. It enables considerable enlargement of the RNA set to be envisaged and analysed as highlighted in Figure 6.

Novel filtering options, resulting from the introduction of more conformational parameters to the database, have been implemented. This has allowed us to adjust the search engine so that it can address a variety of the information stored in RNA FRABASE 2.0.

### Improvements to the web interface

**Figure 7:**
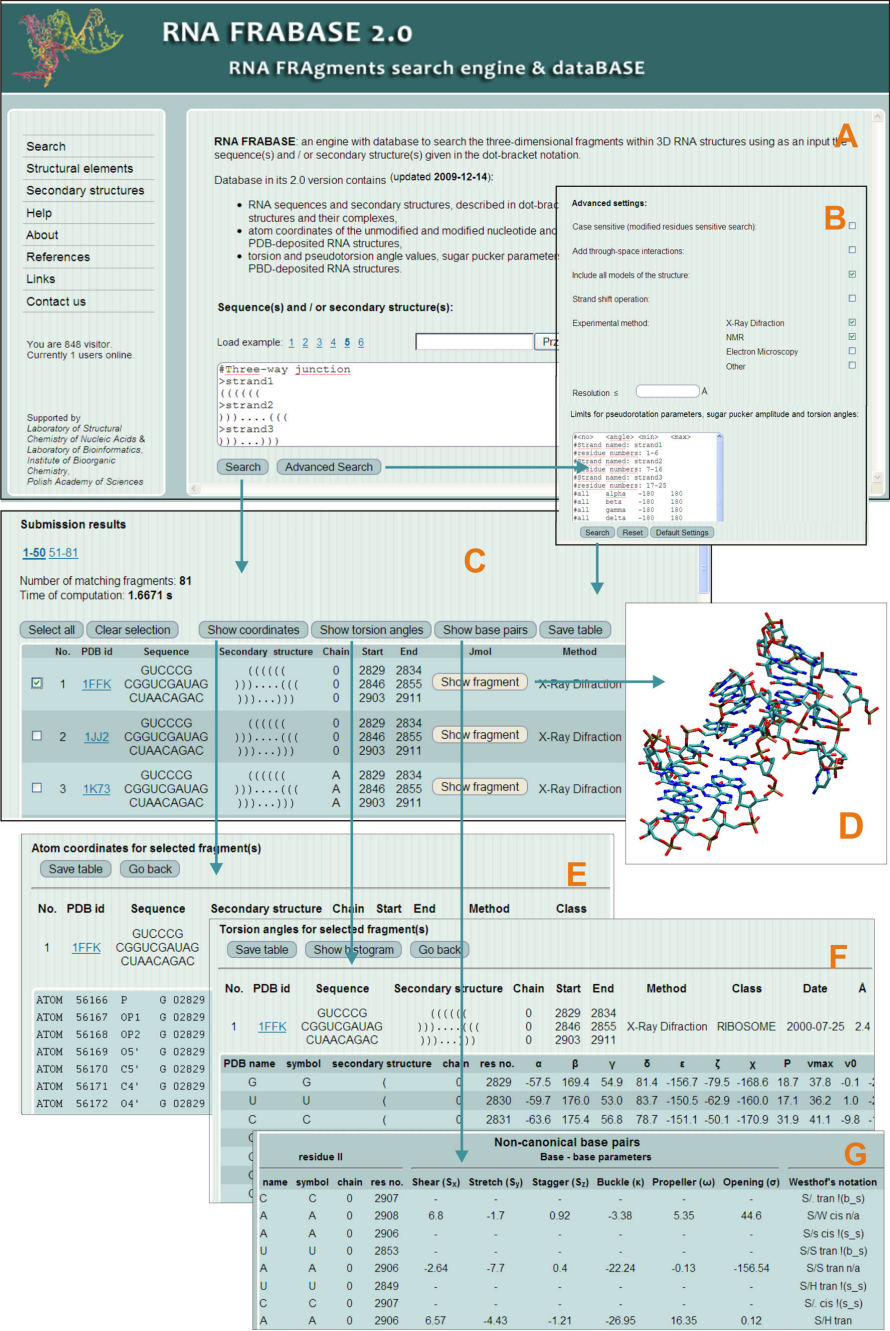
**RNA FRABASE 2.0 interface snapshots concerning the "Search" menu**. Panel A shows the front page window. Panel B enables 'Advanced search' facilities with the provided filters. Panel C illustrates a list of query-matching RNA fragments with related structural information. Panel D highlights the Jmol visualization of the selected fragment. Panels E, F and G are the windows for 'Atom coordinates', 'Torsion angles' and 'Base pairs' for the selected fragment, respectively.

The RNA FRABASE 2.0 web page has a modern outlook (Figure 7). However, the basic layout remains unchanged so that we do not complicate the work of many regular visitors (over a thousand visitors from over 75 countries world wide use RNA FRABASE 1.0 monthly). The new functionality of the search engine has required the introduction of additional options and menu items to facilitate running novel types of search. Thus, two new items in the main menu have appeared: "Structural elements" and "Secondary structure". The "Structural elements" menu item allows for the very fast screening of basic structural elements. The "Secondary structures" menu item provides a list of RNA secondary structures given in the extended dot-bracket convention and graphic format (see Figure 3). Furthermore, a wide selection of new filtering options have been implemented. They can be set for queries launched from both, the "Search" (Advanced options page) menu and the "Structural elements" (advanced options at the bottom of each element tabsheet) menu. This database expansion has resulted in new ways of presenting results. The tables containing values of torsion angles and base pair classification have been expanded. The list of fragments has been enriched by adding icons of the corresponding RNA 3D structures. For each three-dimensional RNA structure fragment, an instant Jmol [21] visualization has been made available to the user.

### Implementation

RNA FRABASE 2.0 runs in the SUSE Linux environment. It has been developed in the relational management database system PostgreSQL. The search engine is served by the Apache http daemon along with PHP scripts. The web interface has been designed using HTML. The assistant programs have been encoded in AWK and PHP.

## Utility and discussion

RNA FRABASE 2.0 is the only web-accessible tool that enables fast automatic exploration of such a substantial selection of structural parameters available for user-defined 3D RNA fragments. Thus, we believe that our tool can be widely used in the study of RNA features and that it can significantly facilitate the processes for 3D RNA structure modelling. Its superiority over other tools has been demonstrated during the testing phase. The RNA FRABASE engine has been tested for a number of representative 3D RNA motifs, fragments and hypothetical structural elements. These have been selected because of their importance for RNA structure research (e.g. tRNA anticodon loop, acceptor stem, hairpin, decamer duplex). Its performance has been compared to the previous version [12] and also to the recently published FASTR3D [13]. Selected experimental results are presented in Table 3. At the time of the experiments, RNA FRABASE 2.0 contained 1565 RNA structures, while FASTR3D stored 1303 structures derived from PDB. FASTR3D was performed with option 'Match query primary sequence exactly: Yes'. RNA FRABASE 2.0 had the default values of most of the advanced options, except for the cases specified in the description. RNA FRABASE 1.0 was tested with the default settings of all the options.

**Table 3:** RNA FRABASE 2.0, 1.0 and FASTR3D - performance comparison.

Pattern (query)	Description of the query pattern and advanced options (in RNA FRABASE 2.0)	Number of results		
		
		RNA FRABASE 2.0	RNA FRABASE 1.0	FASTR3D
>strand1GAcUgAAgAuc	tRNA anticodon loop (only sequence) default options	61	60	55

>strand1GacUgAAgAuc(.........)	tRNA anticodon loop a) default options b) add through-space interactions: ON	34 47	34 -	32 -

>strand1 NNNGNRANNN(((....)))	Hairpin GNRA loop a) default options b) include all models: ON	2120 2125	1680 -	1498 -

>strand1 NNNGNRANNN((?....?))	Hairpin GNRA loop default options	2392	-	-

>strand1^(((((((((($>strand2^))))))))))$	Decamer duplex default options	67	-	-

>strand1^(((((((>strand2)))))))....$	tRNA acceptor stem default options	143	-	-

>strand1NNNANNN(((.(((>strand2NNNNNN))))))	Duplex with a single adenosine bulge: a) default options b) strand shift operation: ON	356 606	287 -	- -

>strand1(......(>strand2).....)	Internal loop (E loop in 5S rRNA) a) default options b) strand shift operation: ON	258 528	226 -	209 -

>strand1((.......((>strand2))....((>strand3))..))	Three-way junction loop (in ribozyme) default options	12	11	14

>strand1(((....).((>strand2))...((>strand3)).))	Three-way junction loop (in hammerhead) a) default options b) strand shift operation: ON	2 4	2 -	2 -

>strand1(((.[[[[[[)))>strand2(((.]]]]]])))	Kissing loop default options	57	52	48

The superiority of RNA FRABASE 2.0 in the number of results is clearly seen especially when the user searches for small structural elements, larger RNA structures with long sequences and fragments described by the secondary structure only, uses wildcard characters in the query or sets newly added advanced options. As for the processing time, it is comparable to that of FASTR3D and about ten times shorter than RNA FRABASE 1.0.

## Conclusions

In this paper, we have presented RNA FRABASE version 2.0, a web accessible database with an effective search engine, which allows for the automatic search of user-tailored, three dimensional fragments within a set of RNA structures. It provides the user with new structural data and functionalities that are unavailable elsewhere. Its versatility has resulted from the possibility to search for fragments of any size, starting from single residues to very big tertiary structure fragments. It is capable of a variety of major applications to underpin a broad spectrum of RNA research. One can envisage the following major applications of the tool: tailored search of 3D RNA fragments for comparative structure modelling using templates [22], motif search (see [20,23]), extracting conformational data and RNA structure analysis.

## Availability and requirements

RNA FRABASE 2.0 is freely available at http://rnafrabase.cs.put.poznan.pl, implemented in PHP, PostgreSQL and Apache, with all major browsers supported.

## Authors' contributions

MP, MS, JB and RWA conceived of the study and participated in the coordination of the project. MP, MS, JB and RWA participated in the design of the database and the web interface. All authors were involved in algorithm development. MB and SW implemented the search algorithms for small structural elements. MP, MS, MB and RWA contributed to analyse the results. MP, MS, EKB and RWA drafted the manuscript. All authors read and approved of the final draft.
